# 4573 Characterization of vascular disease in an Acta2 mutant mouse model

**DOI:** 10.1017/cts.2020.375

**Published:** 2020-07-29

**Authors:** Anita Kaw, Callie Kwartler, Abhijnan Chattopadhyay, Dianna M. Milewicz

**Affiliations:** 1 University of Texas Health Science Center at Houston

## Abstract

OBJECTIVES/GOALS: *ACTA2* R179 carriers present with early-onset stroke; occlusive lesions of the distal internal carotid artery and branches are filled with cells staining positive for smooth muscle cell (SMC) markers. We will identify pathways leading to increased SMC proliferation and migration and thus occlusion. METHODS/STUDY POPULATION: We generated an *Acta2*^*SMC-R179C/*+^ mouse model, which expresses the *Acta2* R179C mutation in SMCs via the SM22a-Cre-Lox system. rt-PCR performed in aortic tissue confirms the presence of the mutation in the mutant mice and absence in mice with only the floxed allele (WT). We will determine phenotypic differences between mutant and WT brains using micro CT, vascular casting, histology, and immunostaining. We will characterize mutant SMC phenotype in culture by assessing expression of contractile genes and stem cell markers, proliferation, and migration. Single cell RNA (scRNA) sequencing of the brain will assess differential gene expression and cell populations between mutant and WT mice. RESULTS/ANTICIPATED RESULTS: Mutant mice have decreased blood pressure compared to WT mice from 8-24 weeks old, consistent with the phenotype seen in *ACTA2* R179 patients. We expect to see occluded and straighter cerebrovascular arteries and white matter changes in the *Acta2*^*SMC-R179C/*+^ mice. iPSC-derived SMCs from patients show de-differentiation, continued expression of stem cell markers, and increased proliferation and migration. We expect to see a similar phenotype in *Acta2*^*SMC-R179C/*+^ mouse SMCs in culture. Via scRNA sequencing, we expect to see altered transcriptional profiles in mutant mice brains including upregulated proliferative pathways in SMCs, glial cell activation, and gene expression changes in neurons. DISCUSSION/SIGNIFICANCE OF IMPACT: These studies will contribute important information on the pathogenesis of the cerebrovascular disease in *ACTA2* R179 patients. These results may aid in identifying treatments to prevent or decrease risk of developing strokes in those with known predisposition to cerebrovascular occlusive disease.

